# Physical fitness and throwing speed in U13 versus U15 male handball players

**DOI:** 10.1186/s13102-022-00507-0

**Published:** 2022-06-20

**Authors:** Jaime Fernandez-Fernandez, Urs Granacher, Isidoro Martinez-Martin, Vicente Garcia-Tormo, Alba Herrero-Molleda, David Barbado, Juan Garcia-Lopez

**Affiliations:** 1 Faculty of Physical Activity and Sports Sciences, Universidad de León, León, Spain; 2 AMRED, Human Movement and Sports Performance Analysis, Universidad de León, León, Spain; 3 Division of Training and Movement Sciences, Research Focus Cognition Sciences, Faculty of Human Sciences, University of Potsdam, Am Neuen Palais 10, 14469 Potsdam, Germany; 4 Department of Sport Science, Sport Research Centre, Miguel Hernandez University of Elche, Elche, Spain

**Keywords:** Overhead athletes, Shoulder, Injury risk, Sport-specific performance

## Abstract

**Background:**

The aim of this study was to analyze the shoulder functional profile (rotation range of motion [ROM] and strength), upper and lower body performance, and throwing speed of U13 versus U15 male handball players, and to establish the relationship between these measures of physical fitness and throwing speed.

**Methods:**

One-hundred and nineteen young male handball players (under (U)-13 (U13) [n = 85]) and U15 [n = 34]) volunteered to participate in this study. The participating athletes had a mean background of sytematic handball training of 5.5 ± 2.8 years and they exercised on average 540 ± 10.1 min per week including sport-specific team handball training and strength and conditioning programs. Players were tested for passive shoulder range-of-motion (ROM) for both internal (IR) and external rotation (ER) and isometric strength (i.e., IR and ER) of the dominant/non-dominant shoulders, overhead medicine ball throw (OMB), hip isometric abductor (ABD) and adductor (ADD) strength, hip ROM, jumps (countermovement jump [CMJ] and triple leg-hop [3H] for distance), linear sprint test, modified 505 change-of-direction (COD) test and handball throwing speed (7 m [HT7] and 9 m [HT9]).

**Results:**

U15 players outperformed U13 in upper (i.e., HT7 and HT9 speed, OMB, absolute IR and ER strength of the dominant and non-dominant sides; Cohen’s *d:* 0.76–2.13) and lower body (i.e., CMJ, 3H, 20-m sprint and COD, hip ABD and ADD; *d:* 0.70–2.33) performance measures. Regarding shoulder ROM outcomes, a lower IR ROM was found of the dominant side in the U15 group compared to the U13 and a higher ER ROM on both sides in U15 (*d:* 0.76–1.04). It seems that primarily anthropometric characteristics (i.e., body height, body mass) and upper body strength/power (OMB distance) are the most important factors that explain the throw speed variance in male handball players, particularly in U13.

**Conclusions:**

Findings from this study imply that regular performance monitoring is important for performance development and for minimizing injury risk of the shoulder in both age categories of young male handball players. Besides measures of physical fitness, anthropometric data should be recorded because handball throwing performance is related to these measures.

## Background

Handball is an intermittent sport which requires high levels of different physical fitness components such as endurance, strength, speed, and coordination [1]. In terms of performance, handball is characterized by several high-intensity actions during matches, including jumps, accelerations, decelerations, and changes-of-direction [2], interspersed with throwing, hitting, blocking, and pushing actions, and combinations thereof [3]. Thus, the identification of the most important physical fitness measures for successful team handball performance appears to be of great interest for coaches and sport scientists [4]. The respective knowledge will enable the identification of strengths and weaknesses in physical fitness that can individually be addressed through targeted strength and conditioning programs.

The ultimate aim of a handball match is to score more goals than the opponent. Accordingly, the handball throw is a fundamental movement skill that should be already developed during the early stages of long-term athlete development [5]. As with other overhead throws (e.g., baseball pitching or tennis serve), handball throwing is a rapid and complex action including a distinctive whole-body, kinetic-chain proximal to distal motion [6]. It has previously been shown that throw velocity and accuracy represent important factors which are decisive for successful scoring [7]. More specifically, ball-throwing velocity will depend, among other factors, on the player’s ability to accelerate the ball with an overarm or underarm throw [8]. For this purpose, sufficient levels of shoulder strength are needed to accelerate the ball [9]. During a 2 × 25-min game [10], adolescent male handball players perform around 100 passes and 10 shots on the goal. During an average handball training session, players perform between 120 and 150 throws, highlighting the importance of the throwing motion [6].

As a result of the physical demands induced by training and/or competition, handball players are susceptible to a range of injuries including chronic overuse conditions and acute traumatic injuries [11]. Shoulder injuries represent the most frequently reported overuse injury in adolescent handball with an injury incidence ranging between 0.2 and 1.44/1000 handball training/competition hours [12], and a point prevalence of shoulder pain ranging between 7 and 49% [13]. Although several factors (i.e., shoulder mobility, rapid increase in training load, or scapula dyskinesia) have been found to be associated with shoulder injuries [14, 15], shoulder weakness is a prominent risk factor for overuse injuries in youth players [6, 16]. Thus, the inclusion of shoulder mobility (e.g., shoulder range of motion [ROM] tests) and strength tests (e.g., isometric internal/external isometric rotation tests) are fundamental to detect possible muscular imbalances already at an early age.

Previous studies analyzed the relationship between upper and lower limbs muscles’ strength in handball players of different expertise level and sex [3, 8, 17, 18]. Significant correlations (r = 0.5–0.8) were found between throwing velocity and measures of upper body isokinetic torque [19] and one-repetition maximum performance (1-RM) (i.e., bench press, squat) [3, 8, 17]. Ortega-Becerra et al. [4] showed that throwing performance showed moderate and significant correlations (r = 0.3–0.5) with linear sprint times and jump performance (i.e., countermovement jump (CMJ) and jump squat) in a group of under-18 (U18) and under-16 (U16) handball players. More recently, it was shown that anthropometric variables, such as the body mass index, arm span and height were good predictors of throwing speed in U14 and U16 players [20]. To the authors’ knowledge, there is no study available that examined anthropometrics, physical fitness, and sport-specific performance in a large cohort of young male handball players according to age. In addition, knowledge on potential associations between these measures will help to better target performance testing and training.

Thus, the purpose of this study was to analyze the shoulder functional profile (rotation ROM and strength), upper and lower body performance, and throwing speed in U13 versus U15 handball players. In addition, we aimed to examine the relationship between measures of physical fitness and throwing speed in the same cohort. With reference to the relevant literature [16, 21], we hypothesized that differences in shoulder function (ROM and strength) will be observed on both sides that become more prevalent with increasing biological age. Moreover, we also hypothesized that there will be age-specific (U13 and U15) associations between several performance indicators, especially from the upper body (i.e., shoulder ROM and strength, medicine ball throw) and ball velocities in the handball throws [22].

## Methods

### Participants

A convenience sample of 135 young healthy male handball players volunteered to participate in this study. All players were recruited from the youth academy of Abanca Ademar León handball club (León, Spain) and were selected by their respective coaching staff. Teams from this youth handball academy play at top national level. For the purpose of this study, players were grouped into two age groups: U13 (n = 92; age: 13.0 ± 0.6 years; height: 162.3 ± 7.8 cm; body mass: 54.7 ± 11.7 kg) and U15 (n = 43; age: 14.8 ± 0.6 years; height: 170.8 ± 8.3 cm; body mass: 63.3 ± 10.9 kg). The participating athletes had a mean background of systematic handball training of 5.5 ± 2.8 years and they exercised on average 540 ± 10.1 min per week including sport-specific team handball training and strength and conditioning programs. Table 1 shows a typical weekly training content for the different age-categories. None of the players reported a history of any orthopedic problems or limitations during the previous 12 months. Prior to the start of the study, players and their parents/legal guardians were fully informed about the protocol and provided their written informed consent. All methods were carried out in accordance with relevant guidelines and regulations, and the local Institutional Ethics committee from the University of León (ETICA-ULE-012-2020) approved the procedures in accordance with the latest version of the Declaration of Helsinki.

**Table 1:** Typical weekly training content for the different age-categories

Category	Monday	Tuesday	Wednesday	Thursday	Friday	Saturday
U13 (n = 85)	Tec/Tac 90’	NWU 20’ Tec/Tac 60’	Tec/Tac 60’	NWU 20’ Tec/Tac 60’	Tec/Tac 60–90’	FM 50’
U15 (n = 34)	Tec/Tac 90’	S/PT 60’ Tec/Tac 90’	Tec/Tac 90–120’	S/PT 60’ Tec/Tac 60’	Tec/Tac 90’	FM 60’

Tec/Tac: technical and tactical training; NWU: Neuromuscular warm-up (e.g., preventive exercises; plyometrics, speed/agility drills); S/PT: strength and power training; FM: friendly match

### Experimental design

This cross-sectional study was conducted to compare the performance in measures of physical fitness of two different age-categories of handball players (U15 and U13), from a top-level Spanish handball club, following a similar previous experimental design [36]. Testing sessions were undertaken between 4:30 and 7:00 pm, and players were assessed at their regular training facility. The testing took place in an indoor court (temperature, 22.3–24.4 °C; relative humidity, 54.4–61.0%; Kestrel 4000 Pocket Weather Tracker, Nielsen Kellerman, Boothwyn, PA). Four days prior to the start of the study, the participating young athletes were familiarized with the applied procedures and assessment routines. All tests were completed on the same day. Data collected during the familiarization session were used to calculate the between-day intraclass correlation coefficients (ICCs). Participants were asked to refrain from any strenuous physical workout for 24 h before the tests and to be in a fasting state for at least 2 h. Evaluators were experienced strength and conditioning specialists and educated sport scientists who frequently perform the applied tests during regular performance testing throughout the handball season.

### Maturity status

Body height was measured using a fixed stadiometer (± 0.1 cm; Holtain Ltd., Crosswell, UK), sitting height with a purpose-built table (± 0.1 cm; Holtain Ltd., Crosswell, UK), and body mass with a digital scale (± 0.1 kg; ADE Electronic Column Scales, Hamburg, Germany). Pubertal timing was estimated according to the biological maturation of each individual using a predictive equation as described previously [23]. The age of peak linear growth (age at peak height velocity [APHV]) is an indicator of somatic maturity representing the time of maximum growth in stature during adolescence. Maturity offset (MO) was estimated using anthropometric measures included in a regression equation as proposed by Mirwald et al. [24]. Moreover, to account for the reported error, players were grouped into discrete bands based on their MO (pre-PHV [< − 1], circa-PHV [− 0.5 to 0.5], post-PHV [> 1]). Players with a maturity offset ranging between − 1 to − 0.5 and 0.5 to 1 were removed from the dataset (n = 16) [25]. Overall, 119 players were finally included in the study.

### Shoulder range of motion (ROM)

Dominant and non-dominant passive glenohumeral rotation were assessed with a manual inclinometer (ISOMED inclinometer, Portland, Oregon) as previously described [26]. Briefly, each player was lying in supine position on a bench with the shoulder 90° abducted and the elbow flexed at a 90° angle with the forearm perpendicular to the bench. From this starting position, an examiner held the participant's proximal shoulder region (i.e., clavicle and scapula) against the bench to stabilize the scapula by avoiding an overpressure. Another examiner rotated the humerus in the glenohumeral joint to produce maximum passive external (ER) and internal (IR) rotation. Three trials of IR and ER-ROM on each shoulder were performed and the average performance (°) was used for statistical analysis. Moreover, the total ROM (TROM; sum of IR and ER) was calculated. The intraclass correlation coefficient (ICC) and coefficients of variation (CV) measured before the experiment ranged from 0.94 to 0.99 and from 2.1 to 3.5%, respectively.

### Shoulder strength

Isometric shoulder IR and ER strength of the dominant and the non-dominant side were assessed with a portable handheld dynamometer (Nicholas Manual Muscle Tester, Lafayette Indiana Instruments) as previously described [27]. Players were in supine lying position on a plinth with the shoulder abducted at 90° and the elbow flexed at 90°. Three repetitions of 5 s of maximal voluntary effort were performed using a ‘‘make’’ test. Resistance was gradually increased up to maximum without ‘‘breaking’’ the player’s strength. A 10-s resting period was provided between trials. Stability of the upper arm, shoulder, scapula, and trunk were guaranteed through manual fixation of the examiner’s hand, arm, and trunk [28]. The average values were recorded for analysis. In addition, shoulder IR and ER strength performance were normalized to body mass and expressed as N/kg [21]. The ICC and CV ranged from 0.90 to 0.97 and from 4.1 to 5.6%, respectively.

### Overhead medicine ball throw (MBO)

The players stood on a line with their feet side-by-side and slightly apart, facing the direction to which a 2-kg medicine ball had to be thrown. The ball was brought back behind the head and then thrown vigorously forward as far as possible without the player crossing the line. The distance from the line to the point where the ball landed was measured, and the best performance among 3 efforts was recorded to the nearest 5 cm. There was a 45-s rest period between trials. ICC and CV were 0.94 and 4.3%, respectively.

### Handball throwing speed

Before the performance of the throwing test, a standardized warm-up was allowed to the participants which included upper body mobility, active stretching, ballistic exercises and 5 throws performed at progressive velocities. For the 7-m throw test (HT7), 5 throws were performed from the seven-meter line, allowing only one foot to be lifted without stepping on the seven-meter line. By doing so, a penalty throw in handball was simulated, with a 45-s rest between each throw. For the 9-m throw test (HT9), 5 throws were performed after a 3-step running throw from the 9-m line with a vertical jump and a rest of 45-s between throws [29]. The throwing speed was recorded using a high performance sports radar (Stalker Pro 2 Radar Gun, Applied Concepts, Inc./Stalker Radar, Texas, TX, USA) placed 2 m behind the player, and pointing to the executing arm. Only throws that entered directly into the goal, without touching the ground, were considered valid. Molten offcial handballs (Molten Corp., Hiroshima, Japan) were used, (circumference: 50–60 mm; mass: 290–475 g), depending on the regulation size corresponding to the participant’s age. Direct feedback of velocities was provided to encourage maximal effort, and the average speed of the 3 best trials was used for further analysis in both tests (7 and 9 m). For the 7 m throw ICC and CV were 0.90 and 3%, respectively, while for the 9 m throw were 0.92 and 2.9%.

### Hip isometric abductor (ABD) and adductor (ADD) strength

For the measurement of maximal isometric hip ADD and ABD strength in the dominant and non-dominant limbs, a handheld dynamometer (Lafayette Instrument Company, IN, USA) was used which was calibrated prior to each test. The applied test method was in accordance with Thorborg et al. [30]. In brief, the best value from two attempts was used for further analysis from the dominant and non-dominant side. A 30-s rest was allowed between test trials. One experienced examiner performed all tests and provided standardised verbal encouragement during the effort. ICCs and CVs for all tests (ABD and ADD of both, dominant and non-dominant limbs) ranged from 0.92 to 0.97 and 2.9% to 7.5%, respectively.

### Hip range of motion (ROM)

The passive hip IR and ER and passive hip ABD ROMs were measured at an angle of 90° hip flexion of the dominant and non-dominant limbs, using an inclinometer (ISOMED, Portland, Oregon) with a telescopic arm. The applied methods were reported previously [31]. A 30-s rest was granted between trials, limbs, and tests and the best value from two attempts was used for further analysis from the dominant and non-dominant side. Based on the findings from previous studies [31, 32], one or both of the following criteria determined the endpoint for each test: (a) palpable onset of pelvic rotation, and/or (b) the participant feeling a strong but tolerable stretch, slightly before the occurrence of pain. ICC and CV for all tests (IR, ER and ABD of both, dominant and non-dominant limbs) ranged from 0.94 to 0.98 and 1.2% to 7.1%, respectively.

### Countermovement jump (CMJ)

A bilateral CMJ weas performed on a contact-time platform (SportJump System Pro, DSD Sport system, Spain) according to the protocol as described previously by Nuñez et al. [33]. During the jump, hands were held at the hips to minimize the influence of the upper body on jump performance. From a standing position with straight knees, players squatted down to ~ 90° and accelerated at maximal velocity in vertical direction. Each player performed 3 maximal attempts interspersed with 45 s of passive recovery, and the highest jump was recorded and used for statistical analysis. ICC and CV were 0.98 and 2.9%, respectively.

### Triple leg-hop (3H) for distance

The triple leg-hop test requires participants to perform 3 consecutive hops on the same leg aiming for maximum distance [34]. The toes of the participants were positioned immediately behind the zero mark of the measuring tape, and the distance covered was measured as the distance (in m) from the zero mark to the point where the heels touched the ground following the third hop. To be considered a valid attempt, players had to maintain the balance on the tested foot for at least two seconds, before touching the ground with the non-tested foot. Each player performed two attempts with each leg, interspersed with 45 s of passive recovery. The participant’s dominant limb was defined as the preferred stance leg used when the participant kicked a ball as far as possible [34]. Next, the average jump distance for each leg was calculated and used for analysis. ICC and CV were 0.91 and 3.6% for the dominant limb, and 0.84 and 4.3% for the non-dominant limb, respectively.

### Linear sprint test

Time during a 20-m linear sprint (with 5 and 10 m split times) was measured by means of single beam photocell gates placed 1.0 m above the ground level (DSD Sport system, Spain). Each sprint was initiated 1.0 m behind the first photocell gate, which then started a digital timer. Players started the linear sprint test in a standing split position, with their preferred foot behind the starting line, followed by accelerating forward at maximal effort until they passed the last photocell gate placed at 20 m. Each player performed three maximal 20-m sprints with at least 2 min of passive recovery in between the trials [35], and the average performance was calculated. ICC and CV for sprint tests ranged from 0.82 to 0.98 and 1.1% to 3.1%, respectively.

### Modified 505 change-of-direction (COD) test

Players’ capacity to perform a single, rapid 180° change-of-direction over a 5 m distance was measured using a modified version (stationary start) of the 5–0–5 agility test [36]. Players started in a standing split position with their preferred foot behind the starting line, followed by accelerating forward at maximal effort until reaching a line placed at a distance of 5 m. Three trials were completed and the best time was recorded (DSD Sport system, Spain). Two minutes of rest was allowed between trials. The COD deficit (COD_DEF_) for the 505 test was calculated using the following formula: *COD*_*DEF*_ = *(modified 505 time – 10-m time)* [37]. ICC and CV were 0.87 and 1.9% for the dominant side, and 0.86 and 3% for the non-dominant side, respectively.

### Statistical analyses

Group-specific data are presented for each parameter using descriptive statistics (means and standard deviations [SDs]) after normality of data was confirmed using the Kolmogorov–Smirnov test with the Lilliefors’ correction. ANCOVAs were performed to assess between-group differences for each parameter, using AGE as between-subject factor (2 levels: under 13 years old; under 15 years old) and maturity offset (MO) as covariate. ANOVAs instead of ANCOVAs were used when the MO did not show a significant effect as a covariate. Hedges’ *g* index (*d*_*g*_) was used to estimate the effect size of each pairwise comparison [38]. This index is based on Cohen’s *d* [39], but it provides an effect size estimation adjusting for the bias caused by small samples (*n* < 20). According to Cohen [39], effect sizes were categorized as trivial (*d*_*g*_ < 0.2), small (0.2 ≤ *d*_*g*_ <0.5), moderate (0.5 ≤ *d*_*g*_ < 0.8) and large (*d*_*g*_ ≥ 0.8). ANOVAs were performed with the SPSS software package (Version 22.0, IBM, Armonk, NY, USA), establishing significance at *p* < 0.05. For Hedges’ *g*, positive scores indicate that U15 showed better performance than U13. Finally, the Relative Weight Analysis (RWA) [40] was used to examine the relative contribution of each parameter in explaining the variance in handball throwing speed from 7 and 9 m respectively using the RWA Web. RWA allows a better determination of the relative weight of different predictor variables in a multiple regression analysis. Specifically, it reduces the multicollinearity impact based on the calculation of each factor proportional contribution to the criterion variance by adding up both, its direct contribution and its combined contribution with other correlated factors. All potential factors meeting the assumptions of normality and homoscedasticity were entered into the RWA. A backward elimination procedure was used to remove all those parameters that did not influence handball throwing speed (*p* > 0.05) significantly. The relative importance of each factor was calculated as the percentage of the handball throwing speed variance (*R*^2^) that they explained.

Before performing the ANOVAs and RWAs, the sampling software package GPower 3.1. [41] was used to calculate the minimum sample size needed to detect significant results for each statistical analysis. A sample size of 19 participants per age group was found to be necessary to detect large significant main effects of age in ANOVAs (*F* = 0.4; power = 80%; α = 0.05). Taking into account the high heterogeneity shown by PwMS (i.e., large group variance) as well as the high within-subject variability [42, 43], large effect sizes must be observed to find statistically significant between-group differences. Therefore, for pairwise between-group comparisons, a sample size of 21 participants per group was needed to detect large differences between groups (*d*_*g*_ = 0.8; power = 80%; α = 0.05). Finally, regarding the RWA and based on previous results [43], a sample of 61 participants was needed to detect a significant large effect size (*f*^2^ = 0.35; power = 80%; α = 0.05) for the multiple linear regression model with 12 potential predictors.

## Results

Table 2 presents the descriptive variables of players according to their age. Findings show that U15 versus U13 players are significantly taller, heavier, and presented an earlier MO, as well as a later expected APHV.

**Table 2:** Descriptive variables of handball players according to their age

	U13	U15	Age	Effect size
Age (years)	13.0 ± 0.6	14.9 ± 0.5	288.4 (< 0.001)	3.42 (4.02; 2.83)
Body height (cm)	162.0 ± 7.8	172.2 ± 7.1	43.1 (< 0.001)	1.32 (1.76; 0.89)
Body mass (kg)	54.9 ± 11.7	63.6 ± 10.9	14.0 (< 0.001)	0.75 (1.16; 0.34)
APHV (years)	14.0 ± 0.6	14.4 ± 0.6	9.2 (0.003)	0.61 (1.02; 0.21)
MO (years)	− 1.0 ± 0.7	0.6 ± 0.8	104.8 (< 0.001)	2.06 (2.54; 1.59)

One-way independent measures ANOVAs with Age as between subject factor

ANOVA main effects (Age) are presented as F values (p). Effect sizes were calculated as Hedge’s g. Positive effect sizes denote that U15 showed better performances than U13. Effect sizes were presented as means (95% confidence intervals)

U13 = under 13 years; U15 = under 15 years; APHV = age at peak height velocity; MO = maturity offset; cm = centimeters; kg = kilograms

### Group comparisons

For the upper body performance (Table 3), ANOVAs revealed main effects of *age* for both handball throws (HT7, HT9) (53.922 ≤ *F* ≤ 56.673; 1.50 ≤ *d* ≤ 1.53) and medicine ball throw test (OMB) (*F* = 109.513; *d* = 2.13). U15 also showed higher absolute IR and ER strength for the dominant and non-dominant shoulder than U13 (7.532 ≤ *F* ≤ 19.409; 0.56 ≤ *d* ≤ 0.90). However, no *age* effect was observed for the normalized shoulder strength, ER/IR ratio or bilateral strength deficits. Finally, for shoulder ROM, U15 showed lower IR of the dominant arm (*F* = 13.951; *d* = − 0.76) and larger ER ROM in both arms than U13 (23.499 ≤ *F* ≤ 26.062; 0.99 ≤ *d* ≤ 1.04). No *age* effect was observed for the shoulder ER/IR ROM ratio and bilateral ROM deficits.

**Table 3:** Differences between U13 and U15 handball players in upper body performance variables

	U13	U15	AGE	Effect size
OMB (m)	5.9 ± 1.1	8.5 ± 1.4	13.732 (< 0.001)*	2.13 (1.65; 2.62)
HB7 (km·h^−1^)	66.5 ± 7.5	77.0 ± 5.6	1.688 (0.196)*	1.50 (1.05; 1.94)
HB9 (km·h^−1^)	66.3 ± 7.0	76.8 ± 4.6	4.034 (0.047)*	1.53 (1.09; 1.98)
**Shoulder strength**				
Dominant side				
IR (N)	120.3 ± 31.3	137.2 ± 26.1	11.009 (0.001)*	0.56 (0.15; 0.97)
Norm-IR (N/kg)	2.24 ± 0.55	2.19 ± 0.42	0.226 (0.635)	− 0.10 (− 0.50; 0.31)
ER (N)	106.4 ± 28.3	134.2 ± 43.2	3.969 (0.049)*	0.83 (0.42; 1.25)
Norm-ER (N/kg)	1.97 ± 0.47	2.10 ± 0.50	1.596 (0.209)	0.26 (− 0.15; 0.66)
ER/IR ratio	0.91 ± 0.24	0.97 ± 0.21	1.371 (0.244)	0.24 (− 0.16; 0.64)
Non-dominant side				
IR (N)	110.5 ± 28.6	130.6 ± 34.7	12.347 (0.001)*	0.66 (0.25; 1.07)
Norm-IR (N/kg)	2.06 ± 0.47	2.07 ± 0.45	0.010 (0.919)	0.02 (− 0.38; 0.42)
ER (N)	98.7 ± 25.1	126.2 ± 41.2	6.427 (0.013)*	0.90 (0.48; 1.32)
Norm-ER (N/kg)	1.82 ± 0.38	1.97 ± 0.47	0.158 (0.692)*	0.35 (− 0.05; 0.76)
ER/IR ratio	0.91 ± 0.17	0.96 ± 0.15	2.396 (0.124)	0.32 (− 0.09; 0.72)
Bilateral deficit				
IR deficit (%)	7.4 ± 11.7	5.9 ± 14.1	0.334 (0.565)	− 0.12 (− 0.52; 0.28)
ER deficit (%)	6.5 ± 13.4	5.9 ± 10.7	0.053 (0.818)	− 0.05 (− 0.45; 0.35)
**Shoulder ROM**				
Dominant side				
IR (°)	65.7 ± 11.5	57.0 ± 10.7	13.951 (< 0.001)	− 0.76 (− 1.17; − 0.35)
ER (°)	139.6 ± 19.4	159.0 ± 16.0	26.062 (< 0.001)	1.04 (0.62; 1.46)
Non-dominant side				
IR (°)	71.7 ± 11.5	67.3 ± 11.0	0.013 (0.910)	− 0.39 (− 0.79; 0.02)
ER (°)	125.4 ± 17.9	142.3 ± 14.2	23.499 (< 0.001)	0.99 (− 0.57; 1.41)
Bilateral deficit				
IR deficit (°)	− 6.0 ± 10.7	− 10.2 ± 9.8	3.789 (0.054)	− 0.40 (− 0.80; 0.01)

One-way independent measures ANOVA using Age as between subject factor. *One-way independent measures ANCOVAs with Age as between subject factor and MO as covariate. Main effects of age are presented as F values (p). Effect sizes were calculated as Hedge’s g. Positive effect sizes denote that U15 showed better performances than U13. Effect sizes were presented as means (95% confidence intervals)

MBO: overhead medicine ball throw; HB7: 7-m handball throw; HB9: 9-m handball throw; IR: internal rotation; ER: external rotation; Norm-IR: normalized internal rotation strength; Norm-ER: normalized external rotation strength; ROM: range of motion

Results of the lower body performance tests are shown in Table 4. ANOVAs revealed main effects of *age* for most of the analyzed parameters. More specifically, U15 showed higher CMJ values (*F* = 82.328; *d* = 1.86), larger hop distance (3H) (111.034 ≤ *F* ≤ 129.863; 2.15 ≤ *d* ≤ 2.33), faster linear sprint (69.013 ≤ *F* ≤ 73.425; − 1.75 ≤ *d* ≤ − 1.70) and COD times (11.783 ≤ *F* ≤ 19.874; − 0.91 ≤ *d* ≤ − 0.70) than U13. U15 also showed higher hip ABD and ADD strength for both, the dominant and non-dominant lower limbs (26.141 ≤ *F* ≤ 53.490; 1.05 ≤ *d* ≤ 1.50). No main effects of *age* were observed for bilateral hip ABD/ADD strength ratios and COD_DEF_.

**Table 4:** Differences between U13 and U15 handball players in lower body performance variables

	U13	U15	AGE	Effect size
**Lower body performance**				
CMJ height (cm)	23.6 ± 5.0	33.5 ± 5.9	82.328 (< 0.001)*	1.86 (1.39; 2.32)
3H-D (m)	4.28 ± 0.63	5.83 ± 0.74	42.184 (< 0.001)*	2.33 (1.83; 2.83)
3H-ND (m)	4.33 ± 0.68	5.81 ± 0.68	39.370 (< 0.001)*	2.15 (1.67; 2.64)
10-m (s)	1.97 ± 0.13	1.76 ± 0.08	69.013 (< 0.001)*	− 1.70 (− 2.15; − 1.24)
20-m (s)	3.56 ± 0.27	3.13 ± 0.17	5.388 (0.022)*	− 1.75 (− 2.21; − 1.29)
COD (s)	3.00 ± 0.23	2.83 ± 0.13	2.068 (0.153)*	− 0.80 (− 1.22; − 0.39)
CODdef (s)	1.03 ± 0.22	1.07 ± 0.12	1.163 (0.283)	0.22 (− 0.19; 0.62)
**Hip isometric strength**				
Dominant side				
ABD (N)	110.9 ± 31.2	143.2 ± 29.7	0.031 (0.577)*	1.05 (0.62; 1.47)
Norm-ABD (N/kg)	2.6 ± 0.7	3.4 ± 0.7	25.180 (< 0.001)*	1.26 (0.82; 1.69)
ADD (N)	109.4 ± 32.5	149.7 ± 38.1	0.035 (0.853)*	1.17 (0.74; 1.60)
Norm-ADD (N/kg)	2.6 ± 0.8	3.5 ± 0.9	29.602 (< 0.001)*	1.10 (0.67; 1.53)
Non-dominant side				
ABD (N)	108.4 ± 31.5	144.2 ± 32.7	0.106 (0.745)*	1.12 (0.69; 1.54)
Norm-ABD (N/kg)	2.6 ± 0.7	3.4 ± 0.8	33.690 (< 0.001)*	1.14 (0.71; 1.57)
ADD (N)	102.5 ± 25.6	144.6 ± 33.3	1.437 (0.233)*	1.50 (1.05; 1.94)
Norm-ADD (N/kg)	2.4 ± 0.6	3.4 ± 0.8	53.463 (< 0.001)*	1.06 (0.64; 1.49)
Ratio ADD/ABD				
D	1.00 ± 0.19	1.05 ± 0.14	1.653 (0.201)	0.25 (0.16; 0.65)
ND	0.97 ± 0.20	1.01 ± 0.11	0.877 (0.351)	0.20 (0.21; 0.60)

One-way independent measures ANOVA using Age as between subject factor.* One-way independent measures ANCOVAs with Age as between subject factor and maturity offset as a covariate. Main effects of age are presented as F values (p). Effect sizes were calculated as Hedge’s g. Positive effect sizes denote that U15 showed better performances than U13. CMJ: countermovement jump; 3H-D: triple leg hop test with the dominant leg; 3H-ND: triple leg hop test with the non-dominant leg; COD: change of direction test; CODdef: COD deficit; D: dominant side; ND: Non-dominant side

### Relative weight analyses (RWA)

The RWA models explained a large variance (66.1% ≤ R^2^ ≤ 70.2%) in the handball throw speed when the whole sample and the U13 were analyzed (Table 5). However, in the U15 group, RWA models for handball throw speed prediction showed only small to moderate explained variance (HT7 = 37.2%; HT9 = 11.7%). When the whole sample or U13 were considered, several factors were significantly associated with the throw speed. Amongst others, participants’ height, body mass and OMB distance showed the highest contribution (9.2% ≤ R^2^ ≤ 20.2%) to the variation in throwing speed. Lower limbs performance factors (i.e., 20-m test, 3H test, and hip ADD strength) (3.0% ≤ R^2^ ≤ 7.2%) and shoulder strength also contributed to explain throwing variance (3.2% ≤ R^2^ ≤ 8.3%).

**Table 5:** Relative weight analysis with the handball throw from the 7 m and 9 m as the dependent variables and their significant (*p* < 0.05) predictive factors (PF) in male handball players of different ages

		Explained variance (%, adjusted R^2^)										
		Total	1st PF	2nd PF	3rd PF	4th PF	5th PF	6th PF	7th PF	8th PF	9th PF	10th PF
All	HT7m	70.2	Height	OMB	Bmass	20 m	3H_ND_	CMJ	H-ADD_ND_	S-IR_D_	S-ER_D_	H-ABD_ND_
			14.4	11.0	9.3	7.2	6.3	5.7	4.8	4.6	4.1	3.0
	HT9m	66.1	Height	Bmass	OMB	CMJ	20 m	3H_ND_	H-ADD_ND_	S-IR_D_	S-ER_D_	
			14.9	10.0	9.2	7.0	6.7	6.7	4.5	4.0	3.2	
U13	HT7m	69.9	Height	OMB	Bmass	S-ER_D_	20 m	S-IR_D_	H-ADD_ND_	3H_ND_		
			20.2	14.6	9.7	8.3	5.5	4.9	3.8	3.0		
	HT9m	66.1	Height	Bmass	OMB	CMJ	20 m	3H_ND_	H-ADD_ND_	S-IR_D_	S-ER_D_	
			14.9	10.0	9.2	7.0	6.7	6.7	4.5	4.0	3.2	
U15	HT7m	37.2	3H_ND_	ROM-IR_D_	20 m	OMB						
			12.4	8.4	8.3	8.1						
	HT9m	11.7	3H_ND_									
			11.7									

D: dominant leg; ND: non-dominant leg; CMJ: countermovement jump (cm); 3H: triple leg hop test (m); 10-m: 10 m sprint (s); 20-m: 20 m sprint (s); OMB: overhead medicine ball throw (m); HT7m: 7 m handball throw (km/h); HT9m: 9-m handball throw (km/h); S-IR: Shoulder internal rotation strength (N); S-ER: shoulder external rotation strength (N); H-ADD: hip adduction strength (N), H-ABD: hip abduction strength (N)

Regarding the U15 group, participants’ performance during the 3H test (11.7% ≤ R^2^ ≤ 12.4%) was the most important factor in explaining throw speed variance. Shoulder IR strength, OMB distance and linear sprint time over 20-m were also significant factors to explain HT7 variance in the U15 group (8.1% ≤ R^2^ ≤ 8.4%).

## Discussion

The aim of this study was to analyze the shoulder functional profile (rotation ROM and strength), upper and lower body performance, and throwing speed of U13 versus U15 male handball players. Another research goal was to examine the relationship between these physical variables and handball throwing speed. The main results showed that chronologically older (U15) players outperformed younger players (U13) in upper (i.e., HT7 and HT9 speed, OMB, absolute IR and ER strength of dominant and non-dominant sides) and lower body (i.e., CMJ, 3H, 20 m sprint and COD, hip ABD and ADD) performance measures (Tables 2, 3 and 4). Regarding shoulder ROM outcomes (Table 3), a lower IR ROM was found of the dominant side in the U15 group compared to the U13 and a higher ER ROM on both sides in U15. It seems that primarily anthropometric characteristics (i.e., body height, body mass) and upper body strength/power (OMB distance) are the most important factors that explain the throw speed variance in male handball players, particularly in U13 ones (Table 5).

Our findings are mainly in agreement with the literature in regards of the effect of chronological age and/or maturity on physical fitness (i.e., upper and lower body muscle strength/power) and motor skill development (i.e., handball throw) in youth. In this regard, it is not surprising that U15 players outperformed the younger players (U13) in almost all performance measurements, especially in the lower body (Table 4). This is consistent with previous research describing youth athletes from other intermittent sports such as soccer or rugby [44, 45]. Thus, the differences observed in the maturational status and, hence, in anthropometric variables (e.g., height and mass), favored the U15 players (Table 2). In this regard, it has been reported that a more advanced maturational status may lead to greater pubertal gains in body height, mass, absolute, and relative muscle mass. Therefore, these developmentally advanced athletes show, on average, superior muscular (e.g., strength, power, speed, agility) and cardiorespiratory fitness (e.g. endurance) levels [46].

Regarding the upper body characteristics (Table 3), handball players, and overhead throwing athletes in general, are at increased risk of sustaining shoulder injuries as a result of the high forces produced by shoulder muscles which are needed to forcefully accelerate the ball during the throwing motion [12]. Shoulder screening (e.g., strength and ROM testing) is considered to play an important role, not only as a diagnostic tool for injured players, but also to design and develop injury prevention strategies for the shoulder [13]. Glenohumeral strength testing can be useful to monitor the capacity of the shoulder (i.e., detecting IR/ER weakness), especially in the ER, as it has been associated with shoulder injuries in handball players [47]. To the best of our knowledge, only few studies described shoulder strength and ROM reference values for adolescent elite handball players [6, 13, 16]

Our results are in line with previous literature related to research with athletes performing overhead sports [6, 28, 48]. Findings from these studies show that with chronologic age, athletes’ absolute isometric shoulder rotation strength increases on both sides, with side-to-side differences (~ 8% in both ER and IR) that exist from early age on and might be due to hand dominance/non-dominance. Comparing the present values with those from other studies is difficult due to methodological reasons such as the application of different test positions and/or test equipment. Nevertheless, the reported glenohumeral strength values in this study appear to be comparable to those described by Asker et al. [6] but in discrepancy with findings from a recent study with U15 youth handball players [16]. These differences can be related, not only to the testing procedures, but also to the training background, as players from the Achenbach et al. [16] study had an average of 8 years of handball experience, while players in our study had 5.5 years.

Furthermore, U15 players were stronger than U13 players in terms of absolute values (Tables 3 and 4). However, when the data were normalized to body mass, no differences were found when comparing age groups. Since muscular strength increases progressively with both, body mass and height [49], the present results can be considered a normal and expected adaptation, as previously reported in other overhead sports [47, 48]. In this regard, intensive exercise and competition can lead to an unbalanced shoulder function profile, with higher IR strength compared to the ER on the dominant side. Cut-off values reported in previous research were ≤ 75% [11], while our results showed “healthy” average IR/ER ratios of 93% (Table 3). However, when analyzing individual ratios, more than 30 players already showed values below the reported cut-off scores for shoulder injury risk which strengthens the idea that an individual approach should be followed in terms of shoulder profiling in youth handball.

In throwing sports, reduced IR and increased ER ROMs have been reported for the dominant arm of asymptomatic overhead athletes [47, 50, 51]. This is considered as a normal soft tissue and/or bony adaptation to long-term repeated throwing motions [52], and has even been suggested to prevent shoulder injuries. Data from this study are in line with previous research (Table 3), and although values can vary due to measurement positions (e.g., seated vs. supine position on a bench) and/or test protocols (e.g., active vs. passive), our results are consistent with the recommended normative shoulder ROM ratios [11]. Although we did not measure the relationship between shoulder ROM deficits and injury risk, and the available research on this topic showed conflicting results [13, 47], some individual GIRD values in the current study can be considered a marker of injury risk, with bilateral differences exceeding more than 20° in some cases. Thus, the use of active, passive or manual therapy forms of stretching, is still recommended to improve posterior shoulder tightness and overall total range of motion in the short-term for asymptomatic young overhead athletes [53].

The ability to sprint over short distances, as well as to change direction during side-stepping movements is of great importance for achieving higher performance levels, allowing players to be faster during transitions (i.e., between attack and defense phases), and during fast breaks and offensive breakthroughs [4]. Moreover, an improved jumping performance seems to be relevant as competitive demands increase, given the relevance of jumping in handball activities such as throwing and blocking [4, 20]. As previously mentioned, maturational advantages (i.e., body height, mass, absolute, and relative muscle mass) enable U15 versus U13 athletes to produce larger forces and to generate better muscle power, which contributes to sprint faster and jump higher (Table 4) [36]. Results are in line with previous research [4, 20], reporting that players showed greater physical capacities, as their age and performance level increased. In the same line, when analyzing the COD test, and considering MO as covariate, results also indicated significant differences between groups, with older players performing better. These results are contrary to a previous study, using a similar sample from the same handball club [36], as well other studies, showing similar COD performance in different age groups of young athletes (e.g., soccer and tennis) [44, 54]. Since there is a disproportional growth and disruption of motor coordination in complex motor coordination tasks (e.g., agility) at the ages around and after the PHV (e.g., “adolescent awkwardness”) [44], a compromised COD ability could be expected in the U15 players, as previously reported [36]. However, based on the data about the weekly training reported herein, we can speculate with the idea that the higher amount of technical and tactical training in the regular schedule of U15 players, was characterized with exercises emphasizing on the sprints and COD, and may be responsible for the meaningful differences found in the speed-related qualities.

Comparable to speed development, the COD capacity appears to improve from childhood through adolescence [55]. During the circa and post-PHV periods, this improvement seems to be directly related to concomitant increases in sex androgen concentrations (i.e., testosterone, growth hormone), particularly in male athletes [55]. In addition, the participating U15 but not U13 players were already engaged in some basic strength and power training, including two weekly sessions, which further explains better lower limbs performance in the older compared with the younger age group (Table 4). Thus, between-group differences in measures of strength and power cannot only be related to developmental factors such as the growth spurt [56], but also to the positive effects of additional strength and conditioning exercises.

Regarding the COD deficit, results from this study showed no differences between age categories (Table 4), which is contrary to previous studies reporting that faster and more powerful individuals, at both, professional and youth levels, tend to present higher COD deficits when compared with their slower and weaker peers [57, 58]. These differences can be related to the fact that although faster handball players will possibly achieve greater “inertia” during sprints and, therefore, need to apply higher breaking forces over longer ground contact times [57], the distances used in the present study (i.e., modified 505) seem not to be sufficient to achieve considerable velocities.

Lower limb muscle strength and power is a basic requirement to perform explosive actions in intermittent sports (e.g., acceleration, COD) [59]. More specifically, activation of the hip muscles may be an important factor in controlling lower extremity motion during dynamic actions [60]. Results from this study showed that U15 outperformed U13 in hip ABD and ADD strength, in both absolute and normalized values (Table 4). In this regard, the use of normalized strength values, relative to the body mass, may minimize inter-player variability and provide a more accurate approach to compare strength levels between youth athletes of different body sizes [49].

To the authors’ knowledge, there are no studies available that analyzed hip strength in different age groups of handball players. Therefore, we cannot compare our findings with results from previous research. Studies investigating different sport disciplines showed that hip strength deficits appear to impede the ability to eccentrically control sudden, powerful over-stretching of the adductors during lateral stride maneuvers involving abduction and external rotation, resulting in increased susceptibility to groin injuries [59, 61]. We found ADD/ABD ratios between 100–105% and 97–101% on the dominant and non-dominant side, respectively (Table 4). These ratios are significantly higher compared with those values that have previously been reported and associated with and increased groin risk injury (< 80%) [61]. Based on these numbers, 10 players of our study would have an increased risk of sustaining groin injuries In this regard, previous research showed that adductor muscle weakness was associated with a fourfold increase in risk of sustaining acute groin injury in players with otherwise normal strength [62]. Therefore, when taking the results of the literature together with our findings, we recommend implementing preventive strengthening programs for the adductor muscles, particularly if imbalances are observed. This preventive exercise means should help to minimize future groin injuries.

This study revealed large explained variance for throwing speed for the whole sample of handball players (7 m handball free throw: 70.2%) and for the U13 age group (7 m handball free throw: 69.9%) (Table 5). A perfect prediction model for a complex action like the present analyzed throws (i.e., 7 and 9 m) seems to be very difficult to obtain since individual technical skills, as well as coordination are also important factors in producing high ball speeds [63]. Anthropometric factors such as body height and mass make the largest contributions to the explained variance. In terms of physical fitness measures, 20-m linear sprint speed and shoulder external rotation strength appear to contribute to handball throw performance. These findings indicate that primarily developmental factors (height, mass) that cannot be manipulated through exercise determine handball throw performance. Notably, in U15 athletes, these anthropometric factors appear not to play a role anymore which is why overall explained variance decreases in U15 compared with U13. In other words, measures of physical fitness become more prevalent in U15 and appear to have larger potential to further improve handball throw performance. In this regard, since U15 players are in maturation stages around or PHV, this would be a key period for strength/power development, which continues throughout adulthood. Thus, the inclusion of well-designed strength training programs aiming to achieve morphological adaptations can lead to obtain positive benefits not only on strength measures, but also on secondary outcomes including linear sprint, agility/change of direction, and sport-specific performances [64]. Moreover, specific trunk muscle and shoulder strengthening programs should be applied to allow proximal stability of the trunk which is a prerequisite for distal mobility of the arm to transfer forces from the lower limbs through a stable trunk to the shoulder and arm muscles [65].

We must acknowledge some limitations of the current study, including the lack of a larger sample of young athletes, especially in the U15 group, or having more distinct age groups to examine the evolution of these parameters throughout the players’ development. The observed findings on the variance of throw speed could be related to lower body performance (e.g., mechanical power in the triple extension during squat or jump squat) or core-strength that were not tested. Moreover, the fact that both handball throws are dynamic movements, the use of isometric shoulder strength tests as applied in this study, could be a limiting factor due to incongruency of muscle contraction velocity during the handball throw versus the isometric contraction. Finally, findings from this study cannot be translated to female handball players or younger/older age groups. Accordingly, more research is needed to verify our findings in these populations.

## Conclusions

In summary, this study revealed that chronologically older (U15) male handball players outperformed their younger counterparts in most measures of physical fitness. In U13 players, handball throw variance was explained by anthropometric and selected physical fitness factors. In U15 players, variance was lower and included only factors of physical fitness. Findings from this study imply that regular performance monitoring, including an individual shoulder profiling (i.e., glenohumeral ROM and strength), is important for performance development and for minimizing injury risk of the shoulder in youth male handball players.

## Data Availability

The datasets used and/or analysed during the current study available from the corresponding author on reasonable request.

## References

[1] Madruga-Parera M, Bishop C, Beato M, Fort-Vanmeerhaeghe A, Gonzalo-Skok O, Romero-Rodríguez D (2019). Relationship between interlimb asymmetries and speed and change of direction speed in youth handball players. J Strength Cond Res.

[2] Hermassi S, Schwesig R, Wollny R, Fieseler G, Van Den Tillaar R, Fernandez-Fernandez J (2018). Shuttle versus straight repeated-sprint ability tests and their relationship to anthropometrics and explosive muscular performance in elite handball players. J Sports Med Phys Fitness.

[3] Marques MC, van den Tilaar R, Vescovi JD, Gonzalez-Badillo JJ (2007). Relationship between throwing velocity, muscle power, and bar velocity during bench press in elite handball players. Int J Sports Physiol Perform.

[4] Ortega-Becerra M, Pareja-Blanco F, Jiménez-Reyes P, Cuadrado-Peñafiel V, González-Badillo JJ (2018). Determinant factors of physical performance and specific throwing in handball players of different ages. J Strength Cond Res.

[5] Debanne T, Laffaye G (2011). Predicting the throwing velocity of the ball in handball with anthropometric variables and isotonic tests. J Sports Sci.

[6] Asker M, Waldén M, Källberg H, Holm LW, Skillgate E (2020). Preseason clinical shoulder test results and shoulder injury rate in adolescent elite handball players: a prospective study. J Orthop Sports Phys Ther.

[7] Raeder C, Fernandez-Fernandez J, Ferrauti A (2015). Effects of six weeks of medicine ball training on throwing velocity, throwing precision, and isokinetic strength of shoulder rotators in female handball players. J Strength Cond Res.

[8] Granados C, Izquierdo M, Ibañez J, Bonnabau H, Gorostiaga EM (2007). Differences in physical fitness and throwing velocity among elite and amateur female handball players. Int J Sports Med.

[9] Van den Tillaar R (2016). Comparison of range of motion tests with throwing kinematics in elite team handball players. J Sports Sci.

[10] Chelly MS, Hermassi S, Aouadi R, Khalifa R, Van Den TR, Chamari K (2011). Match analysis of elite adolescent team handball players. J Strength Cond Res.

[11] Møller M, Nielsen RO, Attermann J, Wedderkopp N, Lind M, Sørensen H (2017). Handball load and shoulder injury rate: A 31-week cohort study of 679 elite youth handball players. Br J Sports Med.

[12] Laver L, Luig P, Achenbach L, Myklebust G, Karlsson J. Handball injuries: epidemiology and injury characterization: Part 1. In: Handball Sports Medicine. 2018. 10.1007/978-3-662-55892-8_11

[13] Asker M, Waldén M, Källberg H, Holm LW, Skillgate E (2017). A prospective cohort study identifying risk factors for shoulder injuries in adolescent elite handball players: the Karolinska Handball Study (KHAST) study protocol. BMC Musculoskelet Disord.

[14] Wilk KE, Obma P, Simpson CD, Cain EL, Dugas J, Andrews JR (2009). Shoulder injuries in the overhead athlete. J Orthop Sports Phys Ther.

[15] Ellenbecker TS, Cools A (2010). Rehabilitation of shoulder impingement syndrome and rotator cuff injuries: an evidence-based review. Br J Sports Med.

[16] Achenbach L, Laver L, Walter SS, Zeman F, Kuhr M, Krutsch W (2020). Decreased external rotation strength is a risk factor for overuse shoulder injury in youth elite handball athletes. Knee Surg Sport Traumatol Arthrosc..

[17] Chelly MS, Hermassi S, Shephard RJ (2010). Relationships between power and strength of the upper and lower limb muscles and throwing velocity in male handball players. J Strength Cond Res.

[18] Saavedra JM, Kristjánsdóttir H, Einarsson I, Guðmundsdóttir ML, Þorgeirsson S, Stefansson A (2018). Anthropometric characteristics, physical fitness, and throwing velocity in elite women’s handball teams. J Strength Cond Res.

[19] Fleck SJ, Smith SL, Craib MW, Denahan T, Snow RE, Mitchell ML (1992). Upper extremity isokinetic torque and throwing velocity in team handball. J Strength Cond Res.

[20] Saavedra JM, Halldórsson K, Kristjánsdóttir H, Þorgeirsson S, Sveinsson G (2019). Anthropometric characteristics, physical fitness and the prediction of throwing velocity in young men handball players. Kinesiology.

[21] Cools AM, Palmans T, Johansson FR (2014). Age-related, sport-specific adaptions of the shoulder girdle in elite adolescent tennis players. J Athl Train.

[22] Schwesig R, Hermassi S, Wagner H, Fischer D, Fieseler G, Molitor T (2016). Relationship between the range of motion and isometric strength of elbow and shoulder joints and ball velocity in women team handball players. J Strength Cond Res.

[23] Sherar LB, Mirwald RL, Baxter-Jones ADG, Thomis M (2005). Prediction of adult height using maturity-based cumulative height velocity curves. J Pediatr.

[24] Mirwald RL, Baxter-Jones ADG, Bailey DA, Beunen GP (2002). An assessment of maturity from anthropometric measurements. Med Sci Sport Exerc.

[25] Read PJ, Oliver JL, De Ste Croix MBA, Myer GD, Lloyd RS (2018). Landing kinematics in elite male youth soccer players of different chronologic ages and stages of maturation. J Athl Train.

[26] Moreno-Pérez V, Elvira J, Fernandez-Fernandez J, Vera-Garcia F (2018). A comparative study of passive shoulder rotation range of motion, isometric rotation strength and serve speed between elite tennis players with and without history of shoulder pain. Int J Sports Phys Ther.

[27] Gallo-Salazar C, Del Coso J, Barbado D, Lopez-Valenciano A, Santos-Rosa FJ, Sanz-Rivas D (2017). Impact of a competition with two consecutive matches in a day on physical performance in young tennis players. Appl Physiol Nutr Metab.

[28] Cools AM, De Wilde L, van Tongel A, Ceyssens C, Ryckewaert R, Cambier DC (2014). Measuring shoulder external and internal rotation strength and range of motion: Comprehensive intra-rater and inter-rater reliability study of several testing protocols. J Shoulder Elb Surg.

[29] Vila H, Manchado C, Rodriguez N, Abraldes JA, Alcaraz PE, Ferragut C (2012). Anthropometric profile, vertical jump, and throwing velocity in elite female handball players by playing positions. J Strength Cond Res.

[30] Thorborg K, Petersen J, Magnusson SP, Hölmich P (2010). Clinical assessment of hip strength using a hand-held dynamometer is reliable. Scand J Med Sci Sport.

[31] Moreno-Perez V, Gallo-Salazar C, Coso J, Ruiz-Pérez I, Lopez-Valenciano A, Barbado D (2020). The influence of a badminton competition with two matches in a day on muscle damage and physical performance in elite junior badminton players. Biol Sport.

[32] Cejudo A, Sainz de Baranda P, Ayala F, Santonja F (2015). Test-retest reliability of seven common clinical tests for assessing lower extremity muscle flexibility in futsal and handball players. Phys Ther Sport.

[33] Núñez FJ, Santalla A, Carrasquila I, Asian JA, Reina JI, Suarez-Arrones LJ (2018). The effects of unilateral and bilateral eccentric overload training on hypertrophy, muscle power and COD performance, and its determinants, in team sport players. PLoS ONE.

[34] Williams M, Squillante A, Dawes J (2017). The single leg triple hop for distance test. Strength Cond J.

[35] Fernandez-Fernandez J, Granacher U, Sanz-Rivas D, Sarabia Marín JM, Hernandez-Davo JL, Moya M (2018). Sequencing effects of neuromuscular training on physical fitness in youth elite tennis players. J Strength Cond Res.

[36] Fernandez-Fernandez J, Martinez-Martin I, Garcia-Tormo V, Garcia-Lopez J, Centeno-Esteban M, Pereira LA (2020). Age differences in selected measures of physical fitness in young handball players. PLoS ONE.

[37] Nimphius S, Geib G, Spiteri T, Calisle D (2013). “Change of direction deficit” measuement on division I amercian football players. J Aust Strength Cond.

[38] Freeman PR, Hedges LV, Olkin I (1986). Statistical methods for meta-analysis. Biometrics.

[39] Cohen J. Statistical Power Analysis for the Behavioral Sciences. Statistical Power Analysis for the Behavioral Sciences (2nd ed.). Hillsdale, NJ: Erlbaum, 2013. doi:10.4324/978020377158.

[40] Johnson JW (2000). A heuristic method for estimating the relative weight of predictor variables in multiple regression. Multivariate Behav Res.

[41] Faul F, Erdfelder E, Lang AG, Buchner A. G*Power 3: A flexible statistical power analysis program for the social, behavioral, and biomedical sciences. In: Behavior Research Methods. Psychonomic Society Inc.; 2007. 175–91. 10.3758/BF03193146 10.3758/bf03193146 17695343

[42] Feys P, Bibby B, Romberg A, Santoyo C, Gebara B, De Noordhout BM (2014). Within-day variability on short and long walking tests in persons with multiple sclerosis. J Neurol Sci.

[43] Visaria J, Thomas N, Gu T, Singer J, Tan H (2018). Understanding the patient’s journey in the diagnosis and treatment of multiple sclerosis in clinical practice. Clin Ther.

[44] Philippaerts RM, Vaeyens R, Janssens M, Van Renterghem B, Matthys D, Craen R (2006). The relationship between peak height velocity and physical performance in youth soccer players. J Sports Sci.

[45] Condello G, Minganti C, Lupo C, Benvenuti C, Pacini D, Tessitore A (2013). Evaluation of change-of-direction movements in young rugby players. Int J Sports Physiol Perform.

[46] Malina RM, Rogol AD, Cumming SP, Coelho E, Silva MJ, Figueiredo AJ (2015). Biological maturation of youth athletes: Assessment and implications. Br J Sports Med.

[47] Clarsen B, Bahr R, Andersson SH, Munk R, Myklebust G (2014). Reduced glenohumeral rotation, external rotation weakness and scapular dyskinesis are risk factors for shoulder injuries among elite male handball players: a prospective cohort study. Br J Sports Med.

[48] Fernandez-Fernandez J, Nakamura FY, Moreno-Perez V, Lopez-Valenciano A, Coso JD, Gallo-Salazar C (2019). Age and sex-related upper body performance differences in competitive young tennis players. PLoS ONE.

[49] Beunen G, Malina RM, Hebestreit H, Bar-or O (2008). Growth and biologic maturation: relevance to athletic performance. The young athlete.

[50] Andersson SH, Bahr R, Clarsen B, Myklebust G (2017). Preventing overuse shoulder injuries among throwing athletes: a cluster-randomised controlled trial in 660 elite handball players. Br J Sports Med.

[51] Moreno-Pérez V, Moreside J, Barbado D, Vera-Garcia FJ (2015). Comparison of shoulder rotation range of motion in professional tennis players with and without history of shoulder pain. Man Ther.

[52] Ben KW, Kuhn JE, Wilk K, Sciascia A, Moore S, Laudner K (2013). The disabled throwing shoulder: Spectrum of pathology—10-year update. Arthroscopy.

[53] Mine K, Nakayama T, Milanese S, Grimmer K (2017). Effectiveness of stretching on posterior shoulder tightness and glenohumeral internal-rotation deficit: a systematic review of randomized controlled trials. J Sport Rehabil.

[54] Fernandez-Fernandez J, Loturco I, Pereira LA, Del Coso J, Areces F, Gallo-Salazar C, et al. Change of Direction Performance in Young Tennis Players: A Comparative Study Between Sexes and Age Categories. J Strength Cond Res. 2020; Jan9: Online ahead of print. 10.1519/JSC.0000000000003484 10.1519/JSC.0000000000003484 31923020

[55] Lloyd RS, Read P, Oliver JL, Meyers RW, Nimphius S, Jeffreys I (2013). Considerations for the development of agility during childhood and adolescence. Strength Cond J.

[56] Lloyd RS, Oliver JL (2012). The youth physical development model: a new approach to long-term athletic development. Strength Cond J.

[57] Freitas T, Alcaraz P, Bishop C, Calleja-González J, Arruda A, Guerriero A, et al. Change of Direction Deficit in National Team Rugby Union Players: Is There an Influence of Playing Position? Sports. 2018; 7: pii: E2. 10.3390/sports7010002 10.3390/sports7010002 PMC6359010 30577602

[58] Loturco I, Jeffreys I, Abad CCC, Kobal R, Zanetti V, Pereira LA (2019). Change-of-direction, speed and jump performance in soccer players: a comparison across different age-categories. J Sports Sci.

[59] Moreno-Pérez V, Lopez-Valenciano A, Barbado D, Moreside J, Elvira JLL, Vera-Garcia FJ (2017). Comparisons of hip strength and countermovement jump height in elite tennis players with and without acute history of groin injuries. Musculoskelet Sci Pract.

[60] Ryan J, DeBurca N, Mc CK (2014). Risk factors for groin/hip injuries in field-based sports: a systematic review. Br J Sports Med.

[61] Nicholas SJ, Tyler TF (2002). Adductor muscle strains in sport. Sport Med.

[62] Engebretsen AH, Myklebust G, Holme I, Engebretsen L, Bahr R (2010). Intrinsic risk factors for groin injuries among male soccer players: a prospective cohort study. Am J Sports Med.

[63] Serrien B, Baeyens J-P (2018). Systematic review and meta-analysis on proximal-to-distal sequencing in team handball: prospects for talent detection?. J Hum Kinet.

[64] Lesinski M, Herz M, Schmelcher A, Granacher U (2020). Effects of resistance training on physical fitness in healthy children and adolescents: an umbrella review. Sport Med.

[65] Saeterbakken AH, Stien N, Andersen V, Scott S, Cumming KT, Behm DG, et al. The effects of trunk muscle training on physical fitness and sport-specific performance in young and adult athletes: a systematic review and meta-analysis. Sport Med. Jan 21. Online ahead of print. 10.1007/s40279-021-01637-0 10.1007/s40279-021-01637-0 PMC9213339 35061213

